# DNA Methylation, Histone Modifications, and Signal Transduction Pathways: A Close Relationship in Malignant Gliomas Pathophysiology

**DOI:** 10.1155/2012/956958

**Published:** 2012-07-17

**Authors:** Raúl Alelú-Paz, Nadia Ashour, Ana González-Corpas, Santiago Ropero

**Affiliations:** Department of Biochemistry and Molecular Biology, School of Medicine, University of Alcalá, Carretera Madrid-Barcelona Km. 33.6, 28871 Madrid, Spain

## Abstract

Gliomas are the most common type of primary brain tumor. Although tremendous progress has been achieved in the recent years in the diagnosis and treatment, its molecular etiology remains unknown. In this regard, epigenetics represents a new approach to study the mechanisms that control gene expression and function without changing the sequence of the genome. In the present paper we describe the main findings about the alterations of cell signaling pathways in the most aggressive glioma in the adult population, namely, glioblastoma, in which epigenetic mechanisms and the emerging role of cancer stem cell play a crucial function in the development of new biomarkers for its detection and prognosis and the corresponding development of new pharmacological strategies.

## 1. Introduction

The majority of Central Nervous System (CNS) tumors have a glial origin. From a clinical point of view, gliomas can be classified into four grades on the basis of its histology and prognosis, encompassing three different tissue types: astrocytomas (about 70%), oligodendrogliomas (10–30%), and ependymomas (less than 10%). In this clinical scale, glioblastoma (GBM) corresponds to grade IV astrocytoma and represents the most aggressive glioma in the adult population, with a median overall survival between 9 and 12 months after the diagnosis, characterized by rapid growth and diffuse invasiveness into the adjacent brain parenchyma.

## 2. Epigenetic Mechanisms in Normal Cells

Epigenetics can be defined as the study of mechanisms that control gene expression in a potentially heritable way [1]. In humans, the most widely studied epigenetic modification is the methylation of cytosine residues at the carbon 5 position (5 mC) within the CpG dinucleotides [2] mediated by DNA methyltransferases (DNMTs), a family of enzymes that catalyze the transfer of a methyl group from S-adenosyl methionine to the DNA. In mammals, there are three main DNMTs: DNMT1, DNMT3a, and DNMT3b. DNMT1 is the most abundant DNMT in the cell and is transcribed mostly during the S phase of the cell cycle [1]. Its activity is focused on the faithfully preservation of DNA methylation patterns, acting preferably on the hemimethylated DNA generated during semiconservative DNA replication. DNMT3a and-3b are thought to be responsible for establishing the pattern of methylation during embryonic development showing a high expression in embryonic stem cells and a downregulation in differentiated cells, although its function is not only restricted to the novo methylation; both contribute to the methylation of the sites missed by DNMT1 at the replication fork [3].

As we have already mentioned, DNA methylation occurs mainly at CpG dinucleotides, which are not randomly distributed throughout the human genome but are concentrated in regions called CpG islands, preferentially located at the promoter region of about 60% of human genes. These CpG island are usually unmethylated in normal tissues allowing gene expression when the appropriate transcription factors are present. Methylation of promoter CpG islands is associated with a closed chromatin structure and transcriptional silence of the associated genes. Although CpG islands are usually unmethylated in normal tissues, some physiological processes require DNA methylation, such as genomic imprinting, the inactivation of X chromosome in females, the regulation of germline-specific genes, and, finally, in the silencing of tissue-specific genes in cell types in which they should not be expressed [4–6]. Among other mechanisms, gene silencing is carried out by the recruitment of methyl-CpG-binding domain proteins (MBD) that leads the recruitment of histone-modifying and chromatin-remodeling complexes.

Despite of the DNA methylation has been described preferably at CpG islands, this epigenetic mechanism is not exclusive of these regions. CpG island shores and regions of lower CpG density close to CpG islands (~2 kb) are associated with transcriptional inactivation, focusing its activity in the regulation of tissue-specific gene expression. On the contrary, in gene bodies this epigenetic mechanism is common in ubiquitously expressed genes and is positively correlated with gene expression involved in elongation efficiency and prevention of spurious initiations of transcription [1]. Finally DNA methylation is present in repetitive elements in order to protect chromosomal integrity preventing the reactivation of endoparasitic sequences.

Histone modifications are the other major epigenetic modification, which consist in dynamic and reversible posttranslational modifications of the residues at N-terminal tails of histones that are mediated by sets of enzymatic complexes that site-specifically attach or remove the corresponding chemical groups [7]. The core histones H2A, H2B, H3, and H4 group into two H2.A-H2.B dimers and one H3-H4 tetramer to form the nucleosome that is the basic unit of the chromatin. Around the histone octamer, a 147-bp segment of DNA wrapped in 1.65 turns and neighboring nucleosomes are separated by, on average, ~50 bp of free DNA [1].

The histone modifications described so far include acetylation, methylation, phosphorylation, ubiquitination, SUMOylation, and ADP-ribosylation, having a main role in important cellular processes such as DNA repair, DNA replication, alternative splicing, and chromosome condensation. In regard to gene expression regulation, histone modifications have been associated with both transcriptional repression and activation. Histone acetylation results from the balance of the activities of HATs (histone acetyltransferases) and HDACs (histone deacetylases) and in general is associated with a less-condensed chromatin state and transcriptionally active gene status [8], while histone deacetylation increases ionic interactions between the positively charged histones and negatively charged DNA, which yields a more compact chromatin structure and represses gene transcription by limiting the accessibility of the transcription machinery. Histone acetylation also plays an important role in regulation of DNA replication and DNA repair [9]. Histone methylation is regulated by histone methyltransferases (HMTs) and is both associated with transcriptional activation and repression, so gene expression is associated with high levels of trimethylated H3K4, H3K36, and H3K79 and, on the contrary, transcriptional repression is characterized by high levels of H3K9, H3K27, and H4K20 methylation.

These epigenetic modifications do not work alone; histones can be modified at different sites simultaneously, giving rise the cross-talk among the different histone marks. Thus, combination of all these marks in a nucleosome or region together with the DNA methylation pattern specifies chromatin structure and so transcriptional activity.

## 3. Epigenetics in the Human Central Nervous System

Dynamic relationships between epigenetic marks described in the previous section reach the higher levels of complexity in the CNS. The brain develops in a well-programmed order, which begins as a sheet of neural stem cells that lead to the formation of neurons at the embryonic stage and the appearance of glial cells at a later embryonic stage and postnatal period [10]. In both populations, epigenetic marks determine the potential of gene transcriptional activity.

Although the epigenetic mechanisms that regulate the gene expression in the CNS are the same as other organs, the human brain is a complex structure that made it necessary the introduction of new variables in its study, that is, an epigenetic connection to brain anatomy. In this regard, it has been described different epigenetic signatures depending on the brain area analyzed [11], so the DNA methylation patterns vary from one region to another, between cell types and, even, among its different subpopulations (i.e., astrocytes and oligodendrocytes). Moreover, the analysis of the DNA methylation in the human brain could not be restricted to the promoter region of the gene; a recent study suggest the necessity to look beyond promoters, specifically to the intragenic regions and its effects on the gene regulation processes in each cell types and brain regions [12].

Finally, it is important to highlight the role of 5-hydroxymethylcytosine (5 hmC) in the DNA methylation-related plasticity in the human brain. This epigenetic mark derives from an enzymatical modification of 5 mC by Tet family proteins through Fe(II) *α*-KG-dependent hydroxylation [13]. The levels of 5 hmC in the CNS are higher in comparison with other tissues and approximately tenfold greater than those seen in embryonic stem cells [14]; although little is know about the function of this new epigenetic mark, it has been suggested that it plays a critical role in postnatal neurodevelopment and aging, as well as in different human neurological disorders [12].

## 4. The Epigenetics of Malignant Gliomas

The signaling network in cancer follows a pattern of stochastic and complex interactions responsible for different processes that form its pathophysiology. These processes involve genetic and epigenetic changes that disturb the normal function of signal transduction pathways regulating cellular processes such as cell proliferation, adhesion, migration, and differentiation. In recent years, a great number of DNA methylation markers have been identified in cancer through the use of the target candidate gene and whole genome approaches, providing a valuable information about the etiology of cancer, and enabling us to the development of new strategies for assessing cancer risk status, detecting tumors as early as possible, monitoring prognosis, and instituting more accurate tumor staging, along with the monitoring of prevention strategies. Four major cancer clinical areas could benefit from DNA methylation markers: cancer detection, tumor behavior, prediction of response to treatment, and therapies that target methylated tumor suppressor genes.

As other types of tumorigenesis, malignant gliomas involve both activation of oncogenes and inactivation of tumor suppressor genes [15]. In this regard, it has been described different genetic alterations related with GBM involved in several processes as control of cell cycle, growth, apoptosis, invasion, and neovascularization. Although tremendous progress has been achieved in the understanding of the molecular mechanisms involved in the genesis and progression of GBM, its epigenetics regulation remains unclear. Keeping in mind that distinct epigenetic signatures has been associated with different GBM subsets, in this paper we will focus on the epigenetic modifications of those genes that have been traditionally related with the pathophysiology of the disease.

## 5. DNA Hypomethylation in GBM

**Figure 1:**
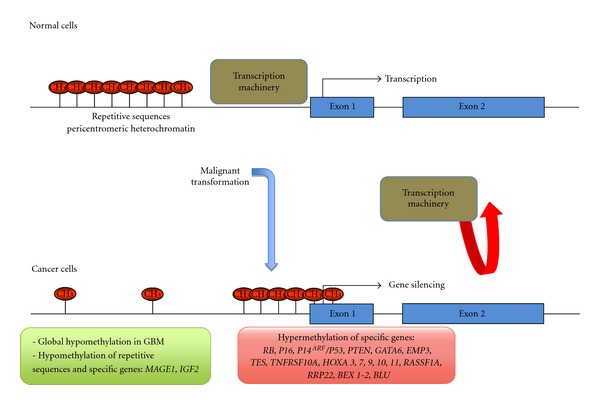
Summary of the changes in the DNA methylation at the repetitive sequences and CpG island associated with GBM.

There are two major DNA methylation phenomena associated with cancer development: hypomethylation and hypermethylation. DNA hypomethylation has been reported mainly in repetitive sequences such as satellite sequences and pericentromeric regions, producing genomic instability and reactivation of transposable elements, events that have been related to the development of several cancers including GBM. Moreover, in a study using a multistage skin cancer progression model, the authors found that DNA hypomethylation is an early event in tumor development, and a biomarker of tumor aggressiveness [16]. In particular, DNA global hypomethylation has been described to occur at high frequency (85%) in primary GBM (Figure 1).

This phenomenon also affects single-copy loci promoting the expression of cancer-related genes such as the melanoma antigen gene (*MAGE1*), that belongs to a group of germline specific genes that become transcriptionally activated in multiple tumors including GBM and low-grade astrocytoma. In these tumors types, *MAGE1* expression has been related with DNA hypomethylation. 

The signal transduction pathway regulated by insulin-like growth factor 2 (*IGF2*) is also dysregulated by DNA hypomethylation. *IGF2* gain of function by loss of imprinting is a common event in several tumor types including GBM. *IGF2* promotes cellular growth through the insulin-like growth factor receptor 1 and phosphoinositide-3-kinase regulating subunit 3 (*PIK3R3*). In particular, IGF2-PIK3R3 signaling pathway promote the growth of a subclass of highly aggressive GBM that lack epidermal growth factor receptor (*EGFR*) amplification [17].

## 6. Promoter Hypermethylation and **** Signal-Transduction Pathways in GBM

Until now, the driving force of DNA methylation research in cancer has been focused on CpG island hypermethylation. In cancer, numerous genes have been identified that have undergone CpG island hypermethylation. These genes include most of the well-established tumor suppressor genes that regulate almost all cellular functions, such as cell cycle (*p*16^*INK*4^, *p*15^*INK*4*b*^, *RB,* and *p*14^*ARF*^), DNA repair (*BRCA1*, *hMLH1*, *MGMT,* and *WRN*), cell-adherence and invasion (*CDH1*, *CDH13*, *EXT1*, *SLIT2,* and *EMP3*), apoptosis (*DAPK*, *TMS1,* and *SFRP1*), carcinogen-metabolism (*GSTP1*), hormonal response (RARB2, ER, PRL, and TSH receptors), and Ras signaling (RASSF1A and NOREIA), microRNAs [18].

In glioma cells, however, gene silencing by DNA hypermethylation can occur at genes that are not expressed in the brain, indicating that not all instances of CpG island hypermethylation are functionally relevant for tumor development and progression. With this consideration, a number of signal-transduction pathways have been found dysregulated by DNA methylation changes in gliomas (Figure 1). For example, promoter hypermethylation of *p*16^*INK*4^, *p*14^*ARF*^, *RB*, *PTEN* and *p53* affects the function of *RB*, *PI3K* and *p53* signaling pathways.

The signaling *p*16^*INK*4^/*RB* pathway is considered one of the most frequently altered in GBM [19]. *RB* is considered as a tumor suppressor gene since functions as inhibitor of cell cycle progression. The *RB *gene product, pRB, has a key role during G1 phase of the cell cycle by binding to the E2F family of transcription factors and generally repressing the target genes by epigenetic mechanism, through the recruitment of corepressor complexes that regulate chromatin structure and function. pRb phosphorylation by mitogen-activated cyclin-dependent kinases (CDKs) impairs the binding to E2F transcription factors and culminate in cell cycle deregulation. pRb also acts in the cell cycle through the inhibition the S phase progression by attenuating cyclin A/Cdk2 activity, resulting in disruption of PCNA function and DNA replication. *RB* gene promoter hypermethylation is the major mechanism underlying loss of *RB* expression in GBM, and this is an early event in tumor progression since *RB* hypermethylation is more frequent in secondary GBM [20]. *p*16^*ink*4^ is located on chromosome 9p21, a region that shows frequent loss of heterozygosity in II–IV gliomas but not in low-grade gliomas. This gene acts as a tumor suppressor gene through its product, *p*16^*ink*4^, that binds to CDK4^2^ and CDK6 to prevent their interaction with cyclin D, keeping RB unphosphorylated and avoiding the cell cycle progression. *p*16^*ink*4^ gene silencing by promoter hypermethylation is also found in gliomas [21].

The cellular signaling regulated by *p*14^*ARF*^/*p*53 is also deregulated by epigenetic mechanism in cancer. *p*53 is a tumor suppressor gene involved in the control of the cell cycle and apoptosis, whose mutation has been described in several neoplasms including GBM. Although the main gene inactivation mechanism for *p*53 is through the mutation plus deletion of this gene, its reduced expression has also been associated with hypermethylation of its promoter region, whereas the inactivation of *p*14^*ARF*^, a stabilizer of *p*53, is mostly associated with DNA methylation rather than mutational activity [22]. The loss of *p*53 function by DNA hypermethylation of itself or *p*14^*ARF*^ produces the loss of cellular response to DNA damage or oncogenic transformation that is mediated by *p*14^*ARF*^/*p*53 signaling pathway.

Other important signal-transduction pathway dysregulated by epigenetic mechanisms is the PI3K/Akt pathway, in which *PTEN* has a main role. The *PTEN* suppressor gene is located at 10q23.3 and encodes a protein with homology to the catalytic domain of tyrosine phosphatases. Its mutations are frequent in primary GBM, and the methylation of its promoter region has been described in different human neoplasms, including GBM [23]. PTEN dephosphorylates phosphatidylinositol (3,4,5)-triphosphate (PtdIns-3,4,5-P3 or PIP-3), an intracellular second messenger related to the activation of Akt pathway. Since AKT pathway is involved in cell growth, cell differentiation and survival, the loss of *PTEN* function by promoter hypermethylation results in increased cell proliferation, cell survival, and tumor invasion [23].

Other genes silenced by promoter hypermethylation and regulating important pathways in cellular homeostasis maintenance have been involved in the processes that underlie the pathophysiology of the GBM (Figure 1). Those genes include* GATA6*, *EMP3*, *TES*, *TNFRSF10A, HOXA*, *RASSF1A, RRP22, BEX1, BEX2, *and* BLU*. *GATA6* is one of the six members of the *GATA* family of transcription factors, which interact with a canonical DNA motif through two highly conserved zinc finger DNA-binding domains [24] with a tissue-specific expression regulating cell-restricted programs of gene expression [25]. Methylation of the *GATA6 *has been involved in different cancer types, as lung and ovarian cancer and, in GBM, is considered as a tumor suppressor gene associated with the formation of the tumor [24].

Epithelial membrane protein 3 (*EMP3*) is a myelin-related gene associated with cell-cell interactions and cell proliferation. *EMP3* promoter has been found hypermethylated and, so, silenced in primary gliomas and neuroblastoma, showing similar features than a tumor suppressor gene [26–29].

*TES* encodes a Sertoli cell secretory protein that contains three LIM domains (double zinc-finger motifs) and mediate protein-protein interactions between transcription factors, cytoskeletal and signaling proteins. It is involved in different processes as cell growth, cell adhesion, and cell spreading acting as a tumor suppressor. Hypermethylation of *TES* in GBM has been described both by pharmacological inhibition of DNA coupled with gene expression microarray profiles and in a microarray-based DNA methylation study [25, 30].

*TNFRSF10A *encodes a protein member of the TNF-receptor superfamily activated by tumor necrosis factor-related apoptosis inducing ligand (TNFSF10/TRAIL), transducing cell death signal, and inducing cell apoptosis. *TNFRSF10A* gene silencing by promoter hypermethylation has been related with osteosarcomas [31], gastric carcinoma [32], and GBM, where it presents a hypermethylation pattern in its promoter region [25, 33].

*HOXA* genes belong to *HOX* gene family that encodes homeodomain-containing transcription factors involved in cell growth, differentiation [34], and embryonic development [35]. Hypermethylation of *HOXA 11* has previously been found in ovarian cancer [36]. In regard with brain cancer, hypermethylation of this gene plus *HOXA 3, 7, 9*, and *10 *has been related with GBM, establishing two of them (9 and 10) as biomarkers for the prognosis of the disease [37].

Ras-Association Domain Family (RASSF) comprises ten members termed RASSF1 to RASSF10. *RASSF1A *encodes a protein similar to the RAS effector proteins required for death receptor-dependent apoptosis, cell-cycle control, and microtubule stabilization. This gene is firmly established as an epigenetically silenced tumor suppressor gene in a wide variety of cancers, including GBM, due to selective CpG methylation of the promoter upstream of the exon encoding the unique N-terminal segment of the isoform [38–40].

Ras-related protein in chromosome 22 (*RRP22*) has been suggested as a candidate tumor suppressor gene in different human cancers, in spite of most of the Ras family members possess oncogenic properties [41]. Expression of *RRP22* is restricted to the CNS [42]; in GBM it is involved in cell growth and apoptosis, suggesting a tumor suppressor role although its relevance and inactivation mechanisms have not been fully assessed so far [41]. A recent study suggests that mRNA *RRP22* levels are decreased in GBM due to the methylation of its promoter region [41].

*BEX 1 and BEX 2 *aremembers of the brain expressed X-linked gene family which have 91% sequence similarity which each other. *BEX 1* encodes a signaling adapter molecule involved in p75NTR/NGFR signaling, playing an important role in cell cycle progression and in the inhibition of neuronal differentiation in response to nerve growth factor. *BEX 2* regulates mitochondrial apoptosis and G1 cell cycle in breast cancer. Both are considered as tumor suppressor genes silenced by methylation of its promoter region in GBM [43].

Finally, hypermethylation of *BLU* gene promoter has been described both for glioma cell lines and astrocytomas [44]. This gene is located immediately centromeric to *RASSF1* locus and contains a predicted MYND domain at its C-terminus essential to the function of many transcription regulatory proteins involved in important transcriptional regulation pathways [44]. In GBM, it has been proposed that *BLU* acts as a tumor suppressor gene in which its hypermethylation together with an unmethylated *CASP8* is associated with prolonged time to tumor progression [45].

Epigenetic changes and in particular DNA methylation are, therefore, as etiologically relevant as the sequences changes that occur via genetic alterations such as point mutations and translocations. Since there is a delicate profile of CpG islands hypermethylation in human tumors, the detection of hypermethylated CpG islands may offer one of the most promising approaches for assessing cancer risk status, to achieve the earliest tumor detection and monitoring prognosis, and to institute more accurate tumor stating, along with the monitoring of prevention strategies. DNA methylation changes have been reported to occur early in the carcinogenesis and, therefore, are potentially good indicators both of existing disease, and even of risk assessment for the future development of disease. Together, these observations encourage us to consider the use of DNA methylation as a therapeutic target for the treatment of cancer. In fact, azacitidine is the first DNA methyltransferase inhibitor to be approved by the US Food and Drug Administration for the treatment of myelodysplastic syndromes.

## 7. DNA Methylation and Resistance to Chemotherapy

The sensitivity of cancer cells to chemotherapy and radiotherapy can be also affected by the epigenetic silencing of different genes. In particular, the reactivation of the silenced suppressor of cytokine signaling I (*SOCS1*) in GBM sensitized these tumors to radiation via inactivation of the *MAPK* pathway [46].

However, the best-known example is the role of promoter hypermethylation of the DNA-repair gene *MGMT* (O^6^-alkylguanine-DNA alkyltransferase) in the response of gliomas to alkylating agents. *MGMT* reverse the alkylation at the O^6^ position of guanine inhibiting the cross-linking of double-stranded DNA induced by alkylating agents such as BCNU (1,3-bis (2-chloroethyl)-1-nitrosourea), ACNU (1-(4-amino-2-methyl-5-pyrimidinyl) methyl-3-(2-chloroethyl)-3-nitrosourea), procarbazine (N-methyl-hydrazine), and temozolomide (TMZ, SCHS2.365) [47, 48]. MGMT mRNA expression varies among different types of gliomas, lacking in approximately 30% of them. This loss of expression is due to *MGMT *promoter hypermethylation [49]. The loss of MGMT expression and function by DNA methylation increases the DNA damage induced by alkylating agents. Thus, *MGMT* methylation increases the sensitivity of GBM patients to alkylating drugs treatment, increasing the overall survival and the time to progression of disease [50]. Interestingly, *MGMT* was found hypermethylated in all long-term survivors (LTS) GBM patients, defined as those patients with a median survival time of more than 3 years [51], proving the study of this gene as a clinically relevant predictor of response to treatment in glioma patients [52]. These results are limited to the adult population in view of the fact that it has been reported the incidence of methylation of *MGMT* promoter in pediatric GBM is rare, losing the prognosis value in this population [53].

## 8. Histone Modifications in GBM

As we have described in a previous section, in addition to DNA promoter hypermethylation, histone modifications are key players in gene expression regulation network. For example, silenced CpG island promoters are characterized by the lost of H3K9 acetylation together with H3K9 methylation. Thus, changes in the histone modifications patterns play a key role in gene expression dysregulation and so in cancer development. The loss of acetylated Lys16 and trimethylated Lys20 of histone H4 is a common event in human cancer [54] that is associated with the hypomethylation of repetitive sequences. These changes can be explained by genetic alterations and/or deregulated expression of genes encoding histone-modifying enzymes. In acute promyelocytic leukemias, for example, a genetic characteristic is the chromosomal translocation that produces the fusion proteins containing RAR-PML and RAR-PLZF. These fusion proteins bind to retinoic acid-responsive elements (RAREs) and recruit the HDAC repressor complex with a high affinity, preventing the binding of retinoic acid, and repressing the expression of genes that regulate normal differentiation and proliferation of myeloid cells. Mutations resulting in altered Class I HDACs expression and activity also occur in cancer. Mutation of the gene encoding HDAC2, for example, occurs in sporadic tumors with microsatellite instability. This mutation produces the loss of HDAC2 expression and activity and leads to a global gene expression deregulation, characterized by the upregulation of transforming genes suggesting a role of HDAC2 mutations in human tumorigenesis [55, 56].

**Figure 2:**
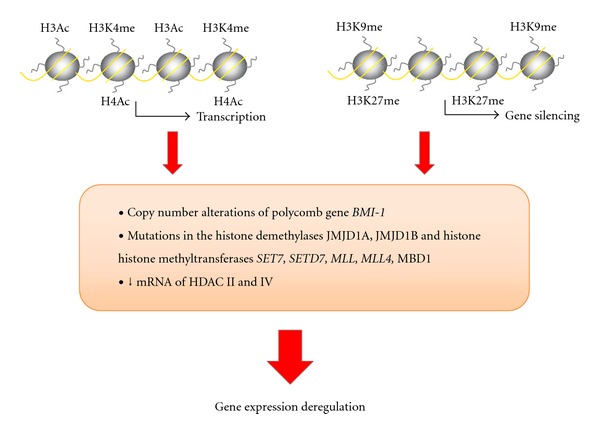
Summary of the alterations in the histone modifiers genes associated with gene expression deregulation in GBM. Ac (acetylation); me (methylation).

Until now there is few evidence for the deregulation of the histone modifying enzymes in GBM (Figure 2). In particular, alteration of the copy number of* BMI-1* gene, that codifies for a protein regulating H3K27 methylation, is a frequent event in low- and high-grade gliomas [57]. Mutations in other histone-modifying enzymes have been found in GBM such as the histone demethylases *JMJD1A* and *JMJD1B*, the histone methyltransferases *SET7*, *SETD7*, *MLL*, *MLL4,* and *MBD1* [58]. Other proteins, as histone deacetylases, are also altered in GBM; class II and IV deacetylases show a decrease in mRNA expression in GBM in comparison with low-grade astrocytomas and normal brain [28]. The coexistence of histone repressive marks and DNA hypermethylation patterns is well represented in one of the genes previously described; methylation of *MGMT* promoter, a frequent event in GBM, is accompanied by H3K9 dimethylation and deacetylation, two other markers of gene silencing [9].

## 9. Future Directions: Cancer Stem Cells and Malignant Gliomas

In the last years, several reports have suggested that an important percentage of the cancer cells within certain tumors have the properties of cancer stem cells (CSCs) [59]. These types of cells have the ability to generate tumors after implantation in animal hosts, to self renew and to give rise to nonstem cells [60]. The cancer stem cell model suggests that the epigenetic changes characteristics of normal stem or progenitor cells are the earliest events in cancer initiation [61]. We can found a link between this event, cancer initiation, and stem cell biology in the function of polycomb (PcG) and trithorax (TrxG) group proteins that provide epigenetic signatures to stem cell identity [62]. These groups affect covalent modifications of histone tails and the position or composition of nucleosomes, as well as DNA methylation, so they have the ability to affect chromatin transcriptional status. In general, PcG repress gene expression, whereas, TrxG proteins activate it. In this regard, the gain of PcG and loss of TrXG in cancer demonstrate the oncogenic and tumor suppressor roles, respectively, of these complexes [63], having many tumors a reactivation in the expression of stem cell-associated genes, such as Hox genes, one of the targets of PcG and TrxG proteins. Unraveling the role of CSCs in the pathophysiology of cancer opens up possibilities for discovering new biomarkers for cancer detection and prognosis and the development of new pharmacological strategies that incorporate agents that target both CSCs and non-CSCs.
